# Supplementary material for: Determining hydrological flow paths to enhance restoration in impaired mangrove wetlands
Source: PLoS One. 2020 Jan 30;15(1):e0227665. doi: 10.1371/journal.pone.0227665 (PMC6992180; doi:10.1371/journal.pone.0227665)
Supplement: S1 Table — The last two columns show the significance of the model and the amount of variance they explain. (DOCX) [file pone.0227665.s001.docx]

|  | Estimate | Std. Error | t-value | *p* | Model statistics | R^2^ adjusted |
| --- | --- | --- | --- | --- | --- | --- |
| Site R - 2013 | 267.75 | 82.01 | 3.26 | 0.002 | *F*_3,30_ = 5.36  ***p* = 0.004** | 0.28 |
| Site R - 2014 | 207.05 | 79.60 | 2.60 | 0.014 |  |  |
| Site R - Rainfall | 0.38 | 0.30 | 1.25 | 0.220 |  |  |
| Site 1 - 2013 | 85.23 | 49.04 | 1.73 | 0.092 | *F*_3,30_ = 2.78  *p* = 0.057 | 0.13 |
| Site 1- 2014 | 110.98 | 45.41 | 2.44 | 0.020 |  |  |
| Site 1- Rainfall | 0.15 | 0.18 | 0.83 | 0.410 |  |  |
| Site 2 - 2013 | -59.13 | 53.31 | -1.10 | 0.275 | *F*_3,31_ = 2.69  *p* = 0.063 | 0.13 |
| Site 2 - 2014 | 10.57 | 51.43 | 0.20 | 0.835 |  |  |
| Site 2 - Rainfall | 0.53 | 0.19 | 2.68 | 0.011 |  |  |
| Site 3 - 2013 | 21.57 | 81.72 | 0.26 | 0.793 | *F*_3,30_ = 3.45  ***p* = 0.028** | 0.18 |
| Site 3 - 2014 | -194.25 | 75.66 | -2.56 | 0.015 |  |  |
| Site 3 - Rainfall | 0.05 | 0.30 | 0.17 | 0.861 |  |  |
| Site 4 - 2013 | 98.95 | 93.03 | 1.06 | 0.296 | *F*_3,30_ = 1.63  *p* = 0.203 | 0.05 |
| Site 4 - 2014 | 135.68 | 86.13 | 1.57 | 0.123 |  |  |
| Site 4 - Rainfall | 0.38 | 0.34 | 1.11 | 0.274 |  |  |
