# Supplementary material for: Determining hydrological flow paths to enhance restoration in impaired mangrove wetlands
Source: PLoS One. 2020 Jan 30;15(1):e0227665. doi: 10.1371/journal.pone.0227665 (PMC6992180; doi:10.1371/journal.pone.0227665)
Supplement: Attachment — Submitted filename: Rebuttal_letter.pdf [file pone.0227665.s002.pdf]

Asesoría Técnica y Estudios Costeros S.C.P.

Especialistas en estudios técnicos y científicos de la

zona costera y áreas de influencia

Mérida, Yucatán, México 10/09/2019

Dear Editors
PLoS ONE

The authors thank the Editor and the reviewers for their review and generous comments on the manuscript.

After a careful revision of the reviewers' comments and suggestions, we include the discussion on how salinity and sulfide concentrations do not yet allow a suitable environment for mangrove settlement and growth. An analysis of some climatic variables and their contribution to hydrological connectivity was added through multiple linear regression models (MLRM), which allowed us to discuss the contribution of precipitation in water levels. We did observe that the effect of the ENSO conditions was neutral through the studied years. Finally, one of the objectives of this study was the inclusion of Fourier analyses to explain climatic and tidal variation; for that reason, on the manuscript, we extended the explanation on the use of this technique.

We believe that our manuscript is now suitable for publication in PLos ONE.

Sincerely,

Arturo Zaldívar Jiménez
Asesoría Técnica y Estudios Costeros S.C.P.

This letter was written on behalf of all authors.

Calle 63 B No. 221 por 122 y 124D, Fraccionamiento Yucalpetén, Mérida, Yucatán.
Teléfono: 999 3716205, Correo electrónico arturo.zaldivar@atecscp.com

Journal Requirements:

Editor: *When submitting your revision, we need you to address these additional requirements. Please ensure that your manuscript meets PLOS ONE's style requirements, including those for file naming. The PLOS ONE style templates can be found at*
Answer: We did make the necessary changes in the manuscript to meet the specifications given in the "Manuscript body formatting guidelines."

Editor: *In your Methods section, please provide additional information regarding the permits you obtained for the work. Please ensure you have included the full name of the authority that approved the field site access and, if no permits were required, a brief statement explaining why.*
Answer: In the new version we added to the methods section the name of the institution responsible for authorizing access to the study sites. Additionally, the authorization letter is attached.

Editor: *We note that you have stated that you will provide repository information for your data at acceptance. Should your manuscript be accepted for publication, we will hold it until you provide the relevant accession numbers or DOIs necessary to access your data. If you wish to make changes to your Data Availability statement, please describe these changes in your cover letter and we will update your Data Availability statement to reflect the information you provide.*
Answer: We added the URL to the repository to Data Availability statement (https://onedrive.live.com/?id=root&cid=5F15A79926E1BA68).
Editor: *We note that Figures 1 and 2 in your submission contain map/satellite images which may be copyrighted. All PLOS content is published under the Creative Commons Attribution License (CC BY 4.0), which means that the manuscript, images, and Supporting Information files will be freely available online, and any third party is permitted to access, download, copy, distribute, and use these materials in any way, even commercially, with proper attribution. For these reasons, we cannot publish previously copyrighted maps or satellite images created using proprietary data, such as Google software (Google Maps, Street View, and Earth). For more information, see our copyright guidelines:*
Aswer: We are making available the image licence of the used Worldview 2 to make the figures 1 and 2.

Editor: *Please include a copy of Table 1 which you refer to in your text on page 11.*
Answer: We removed the reference to Table 1 from the text because such a table did not exist. We have rewritten the entire paragraph to refer it to Figure 3 for a better understanding of the information.

Response to the comments of Review 1

Review 1: The text referring to the last paragraph (Lines 456-461) does not yet justify the results described in the manuscript. The changes achieved still depend on the progressive rehabilitation of local hydrology and biochemistry. However, there has been a noticeable improvement in salinity and sulfide concentrations, but still at high levels that only favor the existence of stressed and dwarf mangrove forests. The suggestion is that the discussion highlights that the conditions generated after intervention in the channels have not yet formed the suitable environment for the development of a mangrove forest comparable to that of the "R site" forest or the most developed one in the region.
Answer: Thanks for all your comments. As suggested, we enriched the discussion by highlighting that in the study area, there are not yet suitable conditions for the optimal development of mangrove forests. We also included the proper references.

Line 140: Indentation of the first line of the paragraph.
Answer: We edited the sentences as suggested.

Line 151: Please, insert the species author´s name... *Rhizophora mangle* L. *Avicennia germinans* (L.) L. when species name appear for the first time.
Answer: As suggested, we inserted the authors' species names.

Line 158: *A. germinans* instead of full name.
Answer: We edited the species' name as suggested.

Line 160: *A. germinans* instead of full name.
Answer: We edited the species' name as suggested.

Line 161: R. mangle instead of full name.
Answer: We edited the species' name as suggested.

Line 163: The topic (2.2 Hydrological restoration) should be inserted in the subtopic (2.3.1 Excavation of channels).
Answer: We edited the structure of the manuscript to meet the reviewer's suggestions.

Line 166: diameter at breast height (DBH).
Answer: We edited the DBH nomenclature.

Line 170: Please, observe the changes to the order of the sections numbering.
Answer: We did check the order of the section numbering to meet the reviewer's suggestion.

Line 172: millimeter instead of mm.
Answer: We did substitute mm for millimeter.

Line 200: In the topic (2.4 Environmental monitoring), please, improve the text to better explain (R = reference site).
Answer: We have rewritten the paragraph, and we did include additional explanations for more clarity about the R site.

Line 201: Indentation of the first line of the paragraph.
Answer: We edited the line as suggested.

Line 204-207: ind ha-1 instead ind.ha-1.
Answer: We have corrected the nomenclature as suggested.

Line 269: In the sentence: "the data did not meet assumptions of normality." Please, insert "and homocedasticity."
Answer: We inserted the word "homocedasticity" in the sentence as suggested.

Line 274: homocedasticity instead of homocedacity
Answer: We corrected the spelling for homocedasticity.

Line285: Please, write the method used.
Answer: The used method is already described in the seccion 2.4.2 Soil biogeochemistry.
Line 290: 004 masl or 0.04 masl?
Answer: We have corrected the numbers. The correct numbering is 0.037 masl.

Line 318: There is no Fig.4B. I suppose it is Fig.4.
Answer: We edited the reference in the text to Fig 4.

Line 341 and 344: Please, indicate in figures 5 and 6 the meaning of A, B, and C.
Answer: As suggested, we added the explanation of the meaning of A, B, and C in the caption of figures 5 and 6.

Line 349: Please note indentation of the first line of the first paragraph in this topic.
Answer: We edited the line as suggested.

Line 354: Even if salinity values in ppt and PSU are nearly equivalent, please stick with just the term. In fact, salinity is a ratio, which value is dimensionless. There is no need to be follow by any unit.
Answer: Thank you for this observation. We choose to eliminate the units for salinity values.

Line 421: 0.004 or 0.04 masl?
Answer: We edited the number.

Line 435: salt instead spartina.
Answer: We changed the word as suggested.

Line 441: delete “el”.
Answer: We did delete “el”.

Line 447: correct “ha-1”.
Answer: We have corrected the nomenclature as suggested.

Fig. 2 – site 5 seems to be site R. If it is correct, please, indicate it in the figure legend.
Answer: We inserted R instead of 5 in the figure.

Fig. 3 – “month-1” instead “month-1”.
Answer: We edited the number as suggested.

Fig. 4 – In principle, there is no rende if the regression coeficiente are not significant. So, please, remove the trend line where it is applied.
Answer: We removed the regressions lines from the graphics where the coefficient´s significance was less than 0.05.

Fig. 5 – Frequency instead Frecuency.
Answer: We corrected the spelling of frequency.

Fig. 7 – ppt or PSU...?
Answer: We did substitute ppt for PSU.

Fig. 8 – mg L-1 instead mg l-1 ppt or PSU ?
Answer: We corrected the nomenclature as suggested.

Response to comments of Reviewer 2.

Editor: *I find that the article could benefit from a comparison with other studies performing similar assessments of the effectiveness of mangrove restoration, salinity, hydrological connectivity and dredging that the authors have ignored.*
Answer: Thank you for all the comments. We reviewed the suggested articles where we did find information about that restoration of the hydrological connection

through channels, as well as the maintenance of them, allows the survival of the mangrove, so we cite to:

Jaramillo, F., Licero, L., Åhlen, I., Manzoni, S., Rodríguez-Rodríguez, J.A., Guittard, A., Hylin, A., Bolaños, J., Jawitz, J., Wdowinski, S., Martínez, O., Espinosa, L.F., 2018b. *Effects of Hydroclimatic Change and Rehabilitation Activities on Salinity and Mangroves in the Ciénaga Grande de Santa Marta, Colombia*. Wetlands 1–13. https://doi.org/10.1007/s13157-018-1024-7.

We did observe differences (Santa Marta and Laguna de Términos) about the contributions of the water sources to the lagoons. The Cienega Grande de Santa Martha receives inputs of freshwater and sediments from the rivers, which have likely contributed to the dusting of the previously excavated channels. In Laguna de Términos most of the water contributions come from the two inlets that connect the lagoon with the Gulf of Mexico. The observed effect after the opening of the tidal channels was an increase in the ebb and flow of the tides, which allowed the appearing of a hydrological network connected to the excavated channels. Moreover, we did not record the dusting of the channels.

The following paragraph of our manuscript explains this condition:

"The modification of the topography may increase the strength of the tidal ebb and flow as topographic levels are reduced [33]. In Zone II, the hydrological gradient produced by tides and by the internal circulation caused by the wind spontaneously carved secondary tidal channels <1 m in width and <0.5 m in depth that constituted a hydrological network connected to the wider and deeper excavated channels".

Editor: Where is the reference point R? It is not shown on the map. Also, why do you assume that the hydroperiod is the same in that control as in the other sites? A justification is needed.

Answer: We added the point for the R site to the map. The description of the site was also edited in the text for a better understanding.

3. Where are the series of water levels? Why did you choose to do your analysis in the Fourier transform of water levels and not on the water levels themselves? It is not clear.

The Fourier transform and spectral analysis have had an essential use in surface and subsurface hydrology. Spectral methods are also recognized as a critical analysis technique in the study of geophysics, oceanography, and meteorology. We propose this type of analysis to evaluate the hydrological connectivity between the mangrove and the adjacent water body.

The series of water levels were used for spectral analysis. They were not included because the dominant frequency spectra were plotted. In such a way we were able to describe different signals such as seasonality and tide. These phenomena are not evident in the time series or the hydroperiod (level, frequency, duration) alone.

Calle 63 B No. 221 por 122 y 124D, Fraccionamiento Yucalpetén, Mérida, Yucatán.
Teléfono: 999 3716205, Correo electrónico arturo.zaldivar@atecscp.com

The changes in the water levels within the mangrove are modulated by the different phenomena involved in the hydrological regime of Laguna de Términos. For example, the winds, increases in the tides, temperature, and rainfall can be identified when seasonal changes are recorded. The signals of diurnal and semi-diurnal tides are among the most critical short-term variations, and only by using the spectral analysis, it is possible to identify such variations, which can be understood as hydrological connectivity.

Editor: You never say what is the "biogeochemical characterization" that you focused on? and why did you analyze those in particular?
Answer: It is correct; we define what we meant for biogeochemical characterization in the introduction. We also explained the reasons to analyze it.

Editor: Why have the authors not considered that the hydrological connectivity and salinity levels have recovered because of favorable climatic conditions? How has precipitation changed from the period before to the period after the event, and during restoration? In some of the studies mentioned in 1, we see that it is difficult to attribute mangrove restoration only on dredging activities since climate conditions can become favorable afterwards during restoration because of La Nina. This would greatly improve the analysis. Ruling out the effect of climatic variability on the improvement in hydrological connectivity is necessary here.
Answer: We include in the manuscript the monthly rainfall values for 2012, 2013 and 2014. We made multiple generalized linear models that relate precipitation with the duration of the flood to explain the influence of rainfall on hydrological connectivity. The result of this new analysis indicated that there were no significant effects of the rainfall on the flooding duration. So, in the scope of our study, the variation of the rainfall is not a phenomenon that modulates changes in water levels, neither has a contribution to hydrological connectivity. Similarly, according to NOAA (2019, published online: www.ncdc.noaa.gov/sotc/global/201413) neutral ENSO conditions did not contribute to changes in water levels during the study years.

Editor: Nice use of statistics in the article. However, I do not think you can attribute " an increase in the the correlation coefficient (r)" p. 236 to the restoration. I think you may not be able to compare R2 just like that. Again, more precipitation on the last year and more water in the wetland would have increased the hydrological connectivity. Also, I think you cannot compare R2 between different samples in this way. Can you check if there is a particular test to do this, apart from the Wilcox? Specially, since you have too few data points, although I agree that the relationship between flood duration in R and the sites increases in 2014.

Answer: As decribed in response to comment five, by using multiple linear regression models we explained the contribution of rainfall to the hydrological connectivity. This new analysis complements the Wilcoxon tests. The methodological procedure was added to the methods section.

Editor: L. 188 what model?
Answer: We corrected the sentence by including **"**flow path".

Editor: Fig. 2 - It is difficult to see the agreement between the dredged channels and the stream paths based on the DEM. IN fact, some of the channels are going across elevations? Maybe you can put both on the same figure?
Answer: We edited the line representing the stream paths on the DEM for better visualization.
